# Chronic Gastritis in Dermatitis Herpetiformis: A Controlled Study

**DOI:** 10.1155/2012/640630

**Published:** 2012-04-26

**Authors:** Anna Alakoski, Teea T. Salmi, Kaisa Hervonen, Hannu Kautiainen, Maarit Salo, Katri Kaukinen, Timo Reunala, Pekka Collin

**Affiliations:** ^1^Department of Dermatology, Tampere University Hospital, 33521 Tampere, Finland; ^2^School of Medicine, University of Tampere, 33014 Tampere, Finland; ^3^Unit of Family Practice, Central Finland Central Hospital, and Unit of Primary Health Care, Kuopio University Hospital, 70211 Kuopio, Finland; ^4^Valkeakoski Regional Hospital, Department of Medicine, 37600 Valkeakoski, Finland; ^5^Department of Gastroenterology and Alimentary Tract Surgery, Tampere University Hospital, P.O. Box 2000, 33521 Tampere, Finland

## Abstract

*Background and Objective*. Previous small studies suggest that chronic atrophic gastritis is common in dermatitis herpetiformis (DH). We here examined the frequency and topography of chronic gastritis in 93 untreated DH subjects and in 186 controls with dyspepsia. *Methods*. Specimens were drawn from the gastric corpus and antrum and examined for atrophy, intestinal metaplasia, and *Helicobacter pylori*. Duodenal biopsies were taken. *Results*. Atrophic corpus gastritis was more frequent in DH than in controls (16.0% and 2.7%, resp., *P* < 0.001); atrophy in the antrum was rare in both groups (3.2% and 1.1%, *P* = 0.34). Intestinal metaplasia was present in 13 (14.0%) DH and 12 (6.5%) control patients (*P* = 0.038) and *H. pylori* in 17 (18.3%) and 17 (9.3%) (*P* = 0.028), respectively. Small-bowel villous atrophy was seen in 76% of the DH patients, equally in patients with and without chronic gastritis. One DH patient with atrophic gastritis developed gastric cancer. *Conclusion*. In DH, chronic atrophic gastritis was common in the corpus, but not in the antrum. *H. pylori* will partly explain this, but corpus atrophy is suggestive of an autoimmune etiology. Atrophic gastritis may increase the risk of gastric cancer. We advocate performing upper endoscopy with sufficient histologic samples in DH.

## 1. Introduction

The majority of patients with dermatitis herpetiformis (DH) evince small-bowel mucosal damage or inflammation similar to that in classic or early-stage celiac disease [1, 2]. Patients rarely suffer from abdominal symptoms, and irrespective of small-bowel mucosal morphology, the rash in DH responds to a gluten-free diet, the treatment of choice for the condition [3, 4]. Coeliac disease and DH share a similar genetic background and occur frequently in the same families; even identical twins may have different phenotypes [5, 6]. Tissue-type transglutaminase is the major autoantigen in coeliac disease, but an immune response to epidermal transglutaminase is probably essential for the development of DH, although this is not fully proven [7, 8]. Altogether, DH is indisputably an extraintestinal manifestation of coeliac disease.

Again similarly to coeliac disease, autoimmune conditions occur together with DH [9]. Earlier studies indicate that chronic atrophic gastritis (CAG) is common in DH and may be of autoimmune origin, but the data are based on a limited number of patients only [10–12]. *Helicobacter pylori *infection is the main agent causing chronic gastritis [13, 14], but autoimmune gastritis may also occur. *H. pylori* gastritis is often patchy and affects the antral mucosa, whereas autoimmune gastritis occurs typically in the corpus of the stomach.

The Sydney System is a systematic approach to determine the topography, morphology, etiology, and severity of gastritis [15]. It has not previously been applied in DH. Small-intestinal biopsy helps to estimate the severity of villous atrophy but is not necessary for the ultimate diagnosis of DH. Provided that CAG is common in patients with DH, this would constitute a further indication for endoscopy. In the present study we examined the occurrence of CAG and *H. pylori*, as classified by the Sydney System, in a large series of DH patients sampled over the past 20 years. The histologic data were compared to those from patients of similar sex and age who were suffering from dyspepsia.

## 2. Material and Methods

The study was carried out over the period 1990–2009 at the Department of Dermatology, Tampere University Hospital. The cohort comprised 93 patients with DH, from whom biopsy samples had been taken from duodenum and from stomach for the classification of gastritis according to the Sydney System. The diagnosis of DH was based on the typical clinical picture and direct immunofluorescence showing granular IgA deposits in the papillary dermis in the uninvolved skin [16]. Patients' medical records were examined. Duodenal biopsy specimens were graded as subtotal villous atrophy, partial villous atrophy, and normal mucosa. Gastric mucosal atrophy was graded from 0 to 3, grade 0 indicating normal morphology and 3 the most severe involvement, in line with the Sydney System [15]. *H. pylori* was not graded, since a positive finding anywhere in the stomach was considered diagnostic for the infection. The patients with DH were regularly followed up in the special outpatient clinic for 1-2 years [17]. A questionnaire was sent to all DH patients who were alive in 2011, and it included questions on adherence to the gluten-free diet, the use of dapsone, and the occurrence of associated diseases and malignancies.

The control group comprised patients suffering from dyspepsia and undergoing upper gastrointestinal endoscopy at the Regional Hospital of our catchment area in 2009–2011. Two control patients of similar sex and age (±5 years) and no small-bowel mucosal villous atrophy were chosen for each DH case, the final series thus consisting of 186 control subjects.

The statistical differences between DH and control patients and DH patients with and without CAG were calculated by chi-square test or Fisher's exact test when appropriate. Odds ratios were given with 95% confidence intervals.

The study was based on the case records, and permission to read these was obtained. A statement of the Ethical Committee was not considered obligatory.

## 3. Results

Atrophy of the corpus and intestinal metaplasia were significantly more common in DH than in the control subjects (Table 1). By contrast, there was no significant difference between the groups in the occurrence of antral atrophy, which was altogether a relatively uncommon finding. The mean score for atrophy in the corpus was 1.6 in DH patients and 2.3 in control subjects.

Seven (44%) DH patients with CAG had associated intestinal metaplasia in the body of the stomach and additional two patients in the antrum (Table 2). *H. pylori* infection was significantly more frequent in DH than in controls (18% and 9%, resp., Table 1). One patient (no. 3, Table 2) with pangastritis and intestinal metaplasia in the initial biopsy developed gastric cancer one year later. Forty-four percent of DH patients with CAG showed *H. pylori* in the gastric mucosa, compared to 14% without CAG (Table 3).

Table 3 shows the 16 DH patients with CAG to be older (mean 63 years) than the 78 without (mean 44 years). Small-intestinal villous atrophy was found in 76.6% of patients with DH and was equally common in patients with and without CAG. Thirty percent of patients with DH reported abdominal complaints; again, there was no significant difference between patients with or without CAG (Table 3).

All 16 DH patients with CAG started a gluten-free diet after the diagnosis of DH, nine in addition using daily 25 mg to 50 mg of dapsone to control the rash. All maintained a strict diet and no longer needed dapsone at the end of the followup.

Associated autoimmune diseases were found in three DH patients with CAG; one had hypothyroidism, one pernicious anaemia and Graves' disease, and one vitiligo (Table 2). One DH patient with CAG developed prostate cancer, and two patients had had breast cancer before the diagnosis of DH.

## 4. Discussion

The frequency of CAG in the corpus was significantly more common in the DH patients than in the control subjects suffering from dyspepsia. No such a difference was seen in the antrum of the stomach. Previously, Primignani et al. [12] conducted a study in 57 Italian patients with DH and found a prevalence of CAG of 30%, compared to 15% in non-DH control subjects with dyspepsia (*P* < 0.05). Patients with DH do not usually suffer from dyspepsia, and therefore the control group was not analogous to the study group. Storskrubb et al. [18] carried out esophago-gastroduodenoscopy at random for 1000 Swedish adults. The overall frequency of corpus atrophy was 5% and antrum atrophy 2%. Our data thus indicate that atrophic corpus gastritis is more common in patients with DH than in the population in general.

*H. pylori* infection is common in CAG [13, 14, 19]. In line with this, the present DH patients with CAG had *H. pylori *significantly more often than those without CAG. This may be partly explained by the age difference; *H. pylori *is more common in older people, and our patients with GAG were older that those without (Table 3). However, the presence of *H. pylori *in all DH patients (18.3%) was significantly higher than among dyspeptic control subjects with a similar age distribution (9.1%, Table 1). By comparison, Crabtree et al. [20] examined 58 DH patients in Britain and by serological methods found *H. pylori* IgG antibodies in 63% of patients, this frequency being however only slightly higher than in other dermatological patients.

There are some limitations to the present study. It was based on the case records, and it was not possible to re-read the biopsy samples. Nevertheless, we considered that the activity of gastritis, as defined by the Sydney System, [15] would have been unreliable to analyze here. Circulating parietal cell and intrinsic factor antibodies could not be determined. Our DH patients were recruited during the years 1990–2009, whereas the controls were enrolled later, in 2009–2011. This may have affected the results in that during the last decades, the prevalence of *H. pylori* has decreased in Finland and elsewhere [21]. However, all but two DH patients with CAG and also patients with *H. pylori* were diagnosed in 2000–2009.

Previous studies have shown that patients with DH similar to those with celiac disease frequently have associated autoimmune conditions such as thyroid diseases and insulin-dependent diabetes mellitus [9, 22, 23]. In the present study two DH patients with CAG had thyroid disease, one of them also pernicious anemia and one patient vitiligo. Due to the limitations of the study, we cannot be sure whether the CAG in the present DH patients was of autoimmune origin. However, this is quite possible judging from the topography of CAG, that is antrum-sparing gastritis [15], and the overall autoimmune nature of DH [23].

CAG is known to be associated with an increased risk of gastric cancers, which seems to decrease after eradication of *H. pylori* [24, 25]. One of our DH patients with CAG developed gastric cancer one year after the diagnosis of DH. Prior to this, he had shown pangastric atrophy and moderate intestinal metaplasia without *H. pylori* infection. The patient had adhered strictly to a gluten-free diet since the diagnosis; the small-bowel mucosa showed no atrophy, and the rash was controlled without dapsone. Similarly, the small-bowel mucosa and the rash in the other DH patients with CAG responded well to GFD treatment. In contrast, CAG persisted in all 4 patients subjected to control biopsies, indicating that CAG in DH does not respond to gluten withdrawal, as also previously documented [26, 27]. Patients with DH run an increased risk of lymphoma [28, 29], but previous large DH studies have not shown any increased risk of gastric or other cancers [28, 30]. Whether persisting CAG in DH involves a risk of gastric cancer is a possibility which should be examined in further studies.

## 5. Conclusion

The present controlled study showed that patients with DH have at the time of the diagnosis a significantly increased frequency of CAG in the corpus but not in the antrum. In addition to *H. pylori *infection, autoimmune mechanisms may be implicated in the development of gastritis. The DH patients here did not present with any specific gastrointestinal symptoms. One DH patient with CAG developed gastric cancer. In untreated DH we recommend upper gastrointestinal endoscopy, upon which biopsy specimens should be taken not only from the duodenum but also from the gastric corpus and antrum.

## Figures and Tables

**Table 1:** Gastric findings in the 93 patients with dermatitis herpetiformis (female 40, median age 48 years; range 7–76) and 186 control patients with dyspepsia (female 80, median age 56 years; range 18–86).

	Dermatitis herpetiformis *n* = 93	Control patients *n* = 186	Odds ratio	*P*-value
Corpus atrophy	15 (16.0%)	5 (2.7%)	6.96 (CI 2.29–25.16)	<0.001
Antrum atrophy	3 (3.2%)	2 (1.1%)	3.07 (CI 0.34–37.16)	0.34
Intestinal metaplasia^1^	13 (14.0%)^2^	12 (6.5%)^3^	2.36 (CI 1.05–5.30)	0.038
Helicobacter pylori^1^	17 (18.3%)	17 (9.1%)	2.22 (CI 1.09–4.54)	0.028

^1^in corpus or antrum.

^2^antrum 8, corpus 8.

^3^antrum 8, corpus 5.

**Table 2:** Gastric and duodenal findings, dapsone, and gluten-free diet (GFD) treatment, and associated diseases and malignancies in 16 dermatitis herpetiformis (DH) patients with chronic atrophic gastritis.

Patient no.	Sex/age (years)	Year of DH diagnosis	Gastric findings			Duodenal histology at diagnosis/on GFD	Associated autoimmune diseases and malignancies
			Corpus atrophy/metaplasia^1^	Antrum atrophy/metaplasia^1^	*Helicobacter pylori*		
1	F/53	1996	1/1	0/0	−	PVA	Breast cancer 1992
2	F/69	1999	1/0	0/0	−	PVA	—
3	M/68	2000	1/2	2/2	−	PVA/N	Gastric cancer 2001
4	M/64	2001	3/2	0/0	−	PVA/N	Hypothyreosis >10 yrs before DH diagnosis Prostate cancer 2010
5	M/61	2001	1/0	0/1	−	N/N	—
6	F/58	2001	1/0	0/0	+	PVA/N	—
7	F/62	2001	2/0	0/0	−	PVA	Breast cancer 1992
8	M/57	2001	1/0	0/0	+	PVA	—
9	M/48	2002	1/0	0/0	+	N	—
10	M/71	2003	2/1	0/0	−	N	Vitiligo 2003
11	F/56	2003	3/1	0/0	−	PVA/N	Pernicious anemia 2004 Graves' disease 2005
12	M/71	2004	2/1	0/2	+	N	—
13	M/76	2006	3/2	0/1	−	N	—
14	F/74	2007	1/0	1/0	+	PVA	—
15	F/63	2007	1/0	0/0	+	SVA	—
16	M/69	2008	0/0	2/2	+	SVA	—

SVA: subtotal villous atrophy, PVA: partial villous atrophy, N: normal mucosa at diagnosis and on a gluten-free diet (GFD).

^1^score 0–3 according to the Sydney System.

**Table 3:** Data on 94 patients with dermatitis herpetiformis. Comparison between cases with and without chronic atrophic gastritis (CAG).

	DH patients with CAG *n* = 16	DH patients without CAG *n* = 78	*P*-value
Mean age at diagnosis years (range)	63.0 (47–76)	43.9 (7–76)	
Men	9 (56.3%)	45 (57.7%)	
Duodenal histology			
(i) partial or subtotal villous atrophy	11 (68.8%)	61 (78.2%) 17 (21.8%)	0.52
(ii) normal mucosa	5 (31.2%)^1^		
*Helicobacter pylori *	7 (44.0%)	11 (14.1%)	0.012
Abdominal complaints	5 (31.2%)	23 (40.4%)^1^	0.36

DH: dermatitis herpetiformis.

^1^Data available on 57 patients.
